# Unraveling Adipose Tissue Dysfunction: Molecular Mechanisms, Novel Biomarkers, and Therapeutic Targets for Liver Fat Deposition

**DOI:** 10.3390/cells13050380

**Published:** 2024-02-22

**Authors:** Marta Lopez-Yus, Carlos Hörndler, Sofia Borlan, Vanesa Bernal-Monterde, Jose M. Arbones-Mainar

**Affiliations:** 1 Adipocyte and Fat Biology Laboratory (AdipoFat), Translational Research Unit, University Hospital Miguel Servet, 50009 Zaragoza, Spain; martalyus@gmail.com (M.L.-Y.); vbernalm@gmail.com (V.B.-M.); 2 Instituto Aragones de Ciencias de la Salud (IACS), 50009 Zaragoza, Spain; 3 Instituto de Investigación Sanitaria (IIS) Aragon, 50009 Zaragoza, Spain; chorndler@hotmail.com; 4 Pathology Department, Miguel Servet University Hospital, 50009 Zaragoza, Spain; 5 General and Digestive Surgery Department, Miguel Servet University Hospital, 50009 Zaragoza, Spain; sborlananson@gmail.com; 6 Gastroenterology Department, Miguel Servet University Hospital, 50009 Zaragoza, Spain; 7 CIBER Fisiopatología Obesidad y Nutrición (CIBERObn), Instituto Salud Carlos III, 28029 Madrid, Spain

**Keywords:** adipose tissue, obesity, NAFLD, biomarkers

## Abstract

Adipose tissue (AT), once considered a mere fat storage organ, is now recognized as a dynamic and complex entity crucial for regulating human physiology, including metabolic processes, energy balance, and immune responses. It comprises mainly two types: white adipose tissue (WAT) for energy storage and brown adipose tissue (BAT) for thermogenesis, with beige adipocytes demonstrating the plasticity of these cells. WAT, beyond lipid storage, is involved in various metabolic activities, notably lipogenesis and lipolysis, critical for maintaining energy homeostasis. It also functions as an endocrine organ, secreting adipokines that influence metabolic, inflammatory, and immune processes. However, dysfunction in WAT, especially related to obesity, leads to metabolic disturbances, including the inability to properly store excess lipids, resulting in ectopic fat deposition in organs like the liver, contributing to non-alcoholic fatty liver disease (NAFLD). This narrative review delves into the multifaceted roles of WAT, its composition, metabolic functions, and the pathophysiology of WAT dysfunction. It also explores diagnostic approaches for adipose-related disorders, emphasizing the importance of accurately assessing AT distribution and understanding the complex relationships between fat compartments and metabolic health. Furthermore, it discusses various therapeutic strategies, including innovative therapeutics like adipose-derived mesenchymal stem cells (ADMSCs)-based treatments and gene therapy, highlighting the potential of precision medicine in targeting obesity and its associated complications.

## 1. Introduction

Adipose tissue (AT) is a highly dynamic and complex component of the human body, pivotal in regulating various aspects of human physiology. Traditionally regarded as a mere fat storage site, AT has emerged as a crucial multi-depot organ, essential in metabolic processes, energy balance, insulin sensitivity, and immune responses. Its role extends beyond simple fat storage, influencing whole-body physiology and contributing to various pathologies, notably obesity and its associated complications like non-alcoholic fatty liver disease (NAFLD) [1].

AT is classified into two primary types: white adipose tissue (WAT) and brown adipose tissue (BAT), each serving distinct functions [2]. WAT is primarily involved in energy storage and is known for its capacity to expand and store lipids. Its distribution varies significantly among individuals and is influenced by genetic and environmental factors. WAT is predominant in subcutaneous depots, but it is also found surrounding internal organs [3]. BAT, on the other hand, is specialized in thermogenesis through uncoupling oxidative phosphorylation [4]. The discovery of beige adipocytes, also known as brite cells, has complicated our understanding of adipose tissue by revealing their ability to switch between energy storage and thermogenic functions, showcasing the plasticity of these cells [5].

The composition of WAT is remarkably heterogeneous, encompassing not only adipocytes but also a rich network of blood vessels, nerve terminals, and various cell types, including immune cells. This cellular diversity contributes significantly to the tissue’s functionality and its role in metabolic processes [6].

WAT’s primary function involves regulating energy balance through lipid storage and mobilization. It actively participates in lipogenesis, the synthesis of lipids, and lipolysis, the breakdown of stored lipids. These processes are intricately regulated and are critical for maintaining whole-body energy homeostasis [7]. Moreover, AT is a vital endocrine organ, secreting various adipokines that regulate metabolic, inflammatory, and immune processes throughout the body. The endocrine function of WAT is closely linked to its metabolic activities, playing a critical role in glucose and lipid homeostasis [8].

However, WAT dysfunction, particularly in the context of obesity, leads to significant metabolic disturbances. In some, but not all, obese individuals, the WAT cannot correctly expand and store excess lipids, resulting in ectopic fat deposition in organs like the liver, contributing to the pathogenesis of non-alcoholic fatty liver disease (NAFLD) [9]. The condition is characterized by an accumulation of fat in the liver not caused by alcohol consumption and can progress to more severe liver diseases. Recent epidemiological data reveal that NAFLD affects more than 70% of the obese subjects, with its prevalence steadily increasing in parallel with the obesity epidemic [10].

Therefore, understanding the complexity and multi-functionality of WAT is crucial in unraveling the pathophysiological mechanisms underlying obesity and its metabolic complications, which are increasing in the population at an alarming rate. This knowledge is essential for developing effective therapeutic strategies targeting WAT dysfunction and preventing associated comorbidities. The following sections of this narrative review will delve deeper into WAT types, composition, metabolic functions, the causes of WAT dysfunction and its role in liver fat deposition, and the diagnostic approaches and therapeutic strategies for addressing WAT-related pathologies.

## 2. Types and Composition of Adipose Tissue

### 2.1. Types and Location of Adipose Tissue

**Figure 1:**
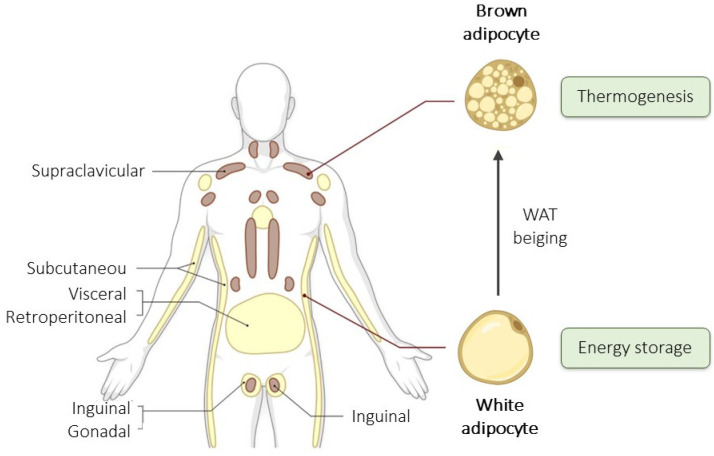
Adipose tissue distribution in humans. White adipose tissue (WAT), represented in yellow, is primarily responsible for energy storage, while brown adipose tissue (BAT), in brown, is a thermogenic tissue. Modified from Torres Irizarry et al. [14].

AT is an integral and versatile human body component, pivotal in metabolic regulation, energy balance, and overall health [1]. This tissue, primarily comprising white adipose tissue (WAT) and brown adipose tissue (BAT), is distinguished by its distinct functions and cellular composition. WAT is primarily responsible for energy storage, while BAT is a thermogenic tissue that generates heat by uncoupling oxidative phosphorylation [2]. Additionally, a third type, beige or brite (brown in white) AT, has emerged, capable of switching between energy storage and thermogenesis depending on physiological demands. Cold exposure and exercise induce WAT beiging to increase thermogenesis, whereas the absence of heat stress or a high-fat diet inhibit this process [11] (Figure 1).

WAT, more abundant than BAT, varies in individual distribution due to genetic and environmental influences. It accounts for a significant proportion of total body weight, ranging from 5 to 60%. WAT is predominantly located in subcutaneous and visceral depots, with smaller amounts found in bone marrow and muscle tissue [2]. Subcutaneous WAT (scWAT) contains over 80% of total body fat, while visceral WAT (visWAT) comprises 10–20% of total body fat in men and 5–10% in women [12].

The metabolic differences between subcutaneous and visceral AT significantly affect overall health. scWAT is generally considered metabolically healthier compared to visWAT [12]. It is more efficient in storing lipids and has a lower association with metabolic diseases [3]. In contrast, visWAT, found around internal organs, is metabolically active and releases fatty acids directly into the portal circulation, leading to insulin resistance and increased risk of metabolic syndrome and cardiovascular diseases. Visceral fat is also more prone to inflammation, contributing to a higher risk of chronic diseases [13].

### 2.2. Composition and Cellular Types of White Adipose Tissue

Most of the WAT volume is represented by mature adipocytes, which are functional cells responsible for energy storage. Adipogenesis is how adipose-derived mesenchymal stem cells (ADMSCs), also present in WAT, differentiate into adipocytes. ADMSCs are crucial for maintaining adipocyte populations and contribute to the tissue’s regenerative capacity [15].

Nevertheless, the cellular composition of WAT extends beyond adipocytes. WAT encompasses various non-adipocyte cell types that include:

- Endothelial cells: Form the lining of blood vessels within AT, playing a crucial role in nutrient transport, angiogenesis, and tissue oxygenation [16].
- Blood cells: Include various types of cells involved in the immune response and the transport of oxygen and nutrients [17].
- Fibroblasts: Contribute to the structural integrity of AT, producing extracellular matrix components that support adipocytes [18].
- Pericytes: Surround endothelial cells, regulate blood flow and capillary stability, and are involved in angiogenesis [19].
- Macrophages: Particularly important in visWAT, these immune cells can contribute to inflammation in AT, especially in the context of obesity and metabolic dysfunction. They play a dual role, maintaining tissue homeostasis and mediating inflammatory responses [20].
- Immune Cells: Including T cells, B cells, and others, they are involved in the immune surveillance of AT and can contribute to inflammation and insulin resistance in obesity [21].

These non-adipocyte cells perform physiological and pathophysiological functions, interacting with adipocytes through secreted factors. The diversity and number of these cell types vary with the development of obesity and metabolic dysfunction [22,23].

## 3. Metabolic Functions and Dysfunctions of Adipose Tisue

### 3.1. Metabolic Functions of White Adipose Tissue 

**Figure 2:**
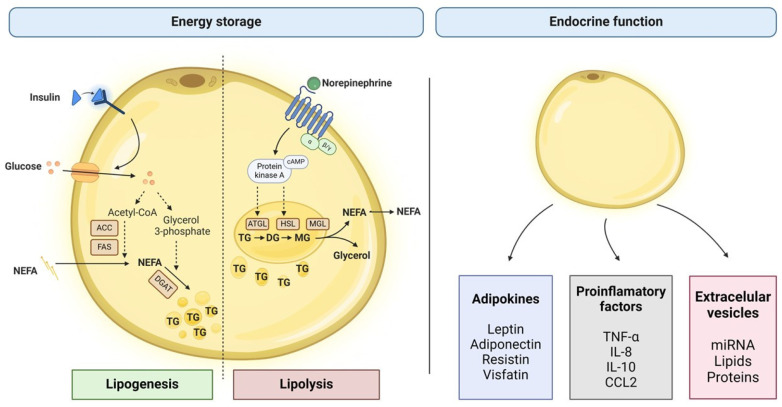
Functions of white adipose tissue (WAT). WAT regulates energy balance by storing (lipogenesis) and releasing (lipolysis) non-esterified fatty acids (NEFA). WAT also secretes a variety of molecules that play essential roles in the regulation of multiple physiological processes. Abbreviation: ACC, acetyl CoA-carboxylase; ATGL, adipose triglyceride lipase; CCL2, CC-chemokin-ligand-2; DGAT, diacylglycerol-O-acyltransferasen; FAS, fatty acid synthase; NEFA, non-esterified fatty acid; HSL, hormone-sensitive lipase; IL-8, interleukin 8; IL-10, interleukin 10; IMGL, monoacylglycerol lipase; TG, triglyceride; and TNF-α, tumor necrosis factor α.

The primary function of WAT is to regulate energy balance by storing and releasing fatty acids (FAs) in response to changes in energy availability. However, WAT also secretes various hormones and cytokines, known as adipokines, that play essential roles in regulating multiple physiological processes [8] (Figure 2).

#### 3.1.1. Lipids Storage and Mobilization

WAT plays a crucial role in regulating energy balance through storing and releasing fatty acids (FAs), a process governed by the dynamic balance between lipogenesis and lipolysis. This balance is essential for maintaining energy homeostasis, particularly during fasting or exercise [24].

Lipogenesis refers to the process of synthesizing new lipids from excess glucose or FAs in the diet. This process occurs in the cytoplasm of adipocytes and is tightly regulated by numerous hormones and enzymes [25]. The primary hormone regulating this process is insulin, which promotes the uptake of glucose and FAs into adipocytes and stimulates the activity of critical enzymes involved in lipid synthesis, such as acetyl CoA-carboxylase (ACC) and fatty acid synthase (FAS) [26].

Lipolysis, on the other hand, refers to breaking down stored lipids in AT to release energy to be used by peripheral organs. Maintaining energy homeostasis during fasting or exercise periods is critical when glucose levels are low and the body needs to rely on stored fat for energy [27]. Lipolysis is regulated by enzymes called lipases. These enzymes are activated by signals from the sympathetic nervous system that are mediated mainly by norepinephrine, but also by epinephrine [28]. The main lipases regulating this process are adipose triglyceride lipase (ATGL), hormone-sensitive lipase (HSL), and monoacylglycerol lipase (MGL) [29].

#### 3.1.2. Endocrine Function

WAT also serves as a vital endocrine organ, secreting a variety of adipokines that regulate metabolic, inflammatory, and immune processes throughout the body [30]. The endocrine function of WAT is regulated by various factors, including nutritional status, physical activity, hormonal levels, and environmental cues. It is closely linked to its metabolic and storage functions, as adipokines are critical in maintaining glucose and lipid homeostasis [31].

Our present knowledge of AT-secreted adipokines includes over 100 proteins exerting cross-talk with other cells/tissues. Leptin and adiponectin are the most abundant and well-characterized adipokines. In contrast, more recently discovered molecules such as resistin, fatty-acid binding protein 4 (FABP-4), omentin, visfatin, lipocalin-2, or chemerin have also been proposed to play important functions [32]. Leptin was the first identified secreted adipokine and has been shown to regulate appetite and energy expenditure. It is an important feedback signal to the brain about the size and status of the AT [33]. On the other hand, adiponectin enhances insulin sensitivity and FAs oxidation in skeletal muscle and liver, which helps maintain glucose and lipid homeostasis [34]. Pro-inflammatory factors such as tumor necrosis factor-α (TNF-α), interleukin 6 (IL-6), interleukin 8 (IL-8), interleukin 18 (IL-18), interleukin 1β (IL-1β), or chemokine ligand 2 (CCL2) are also produced by WAT and are all increased in obesity [35].

### 3.2. Adipose Tissue Dysfunction in Obesity

In the context of obesity, WAT undergoes significant changes. The expansion of adipose depots leads to adipocyte hypertrophy (increase in adipocyte size) and hyperplasia (formation of new adipocytes from precursor differentiation), which contribute to tissue dysfunction [36]. As adipocytes expand in size, their mechanical stress increases because their contact with neighboring cells and extracellular matrix components increases. Adipocytes also experience hypoxia, as the hypertrophic growth of the AT is not accompanied by a similar expansion rate of angiogenesis [37]. Moreover, hypertrophic adipocytes and damaged cells release pro-inflammatory cytokines that attract and activate immune cells. All these promote a chronic low-grade inflammation state in AT that severely alters AT functionality [21].

Furthermore, the enlarged adipocytes in obese individuals present altered lipid metabolism, as they are partially resistant to the antilipolytic effect of insulin. Increased lipolysis leads to increased release of non-esterified fatty acids (NEFAs) into the bloodstream, resulting in the ectopic deposition of lipids, causing lipotoxicity. These changes can contribute to the systemic effects of obesity, including the development of type 2 diabetes, cardiovascular diseases, and certain types of cancer [38,39].

WAT functionality is highly connected to body fat distribution. Excessive calories are primarily stored in scWAT. When scWAT reaches its maximal storage capacity, scWAT becomes dysfunctional and fails to store lipids appropriately, leading to ectopic fat accumulation, primarily in the visWAT and liver [40,41]. Therefore, the distinction between subcutaneous and visceral fat accumulation is crucial, as each fat depot might have a different metabolic function. scWAT is generally considered metabolically healthier, efficiently storing lipids with a lower association with metabolic diseases. In contrast, visWAT, located around internal organs, is metabolically active and linked to increased metabolic syndrome and cardiovascular disease risks [12,13].

Focusing on the differences between adipocytes in subcutaneous vs. visceral adipose tissue, several key aspects emerge:

#### 3.2.1. Adipocyte Size and Number

Adipocytes in scWAT are generally larger and have a greater lipid storage capacity than those in visWAT. The expansion of scWAT occurs primarily through adipocyte hypertrophy, whereas visWAT expansion involves both hypertrophy and hyperplasia [42].

#### 3.2.2. Adipocyte Turnover

The adipocyte turnover rate, including cell death and formation, is higher in visWAT than in scWAT. This dynamic turnover contributes to the overall metabolic activity of visWAT [3].

#### 3.2.3. Insulin Sensitivity

Adipocytes in scWAT are more sensitive to insulin compared to those in visWAT. This difference in insulin sensitivity plays a significant role in the systemic effects of obesity, particularly the development of insulin resistance [43].

#### 3.2.4. Lipolytic Activity

Adipocytes in visWAT exhibit higher lipolytic activity, making them more prone to releasing fatty acids into circulation. This can increase free fatty acid levels in the portal circulation, directly affecting liver metabolism and insulin action [44].

#### 3.2.5. Secretory Profile

The profile of cytokines and adipokines secreted by adipocytes differs between scWAT and visWAT. Adipocytes in visWAT tend to secrete more pro-inflammatory cytokines, contributing to chronic low-grade inflammation, whereas scWAT adipocytes generally have a more benign secretory profile [45].

#### 3.2.6. Oxidative Stress

Adipocytes in visWAT are exposed to higher levels of reactive oxygen species (ROS), which disrupt the redox balance and contribute to inflammatory processes. This impacts visWAT function via mechanisms such as the impairment of adipogenesis or induction of insulin resistance [46].

In summary, the metabolic functions and dysfunctions of adipose tissue, particularly in obesity, are complex and multifaceted. The balance between lipogenesis and lipolysis in adipocytes, the role of adipokines in metabolic regulation, and the differences in metabolic functions between subcutaneous and visceral fat depots are critical in discerning the pathophysiology of obesity and its associated metabolic disorders. Understanding these dynamics is essential for developing effective strategies to manage and treat obesity and its complications.

## 4. Adipose Tissue Expandability and Liver Fat Deposition

### 4.1. Connection between AT Expandability and NAFLD

**Figure 3:**
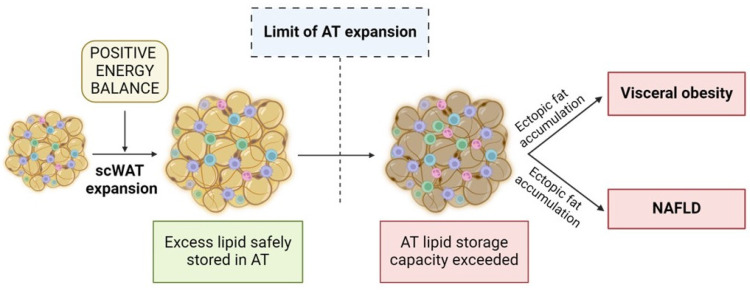
Adipose tissue expansion during obesity. Modified from Vidal-Puig et al. [47]. Abbreviation: AT, adipose tissue; scWAT, subcutaneous white adipose tissue; NAFLD, non-alcoholic fatty liver disease.

The adipose tissue expandability hypothesis is central to understanding the pathogenesis of obesity-related comorbidities, including non-alcoholic fatty liver disease (NAFLD). This hypothesis posits that the body’s ability to store excess calories in scWAT is limited and varies greatly between individuals [9,47]. When scWAT reaches its maximal storage capacity, AT can no longer store lipids effectively, leading to the redirection of lipid flux to other organs. This results in ectopic fat accumulation, primarily in visWAT and the liver, causing insulin resistance and related metabolic complications through lipotoxic and inflammation-driven mechanisms [48,49] (Figure 3).

The imbalance between energy intake and the storage capacity of AT is a crucial factor in the development of NAFLD. Excessive caloric intake, especially when coupled with the limited expandability of scWAT, leads to fat deposition in the liver. This hepatic fat accumulation, or steatosis, is the hallmark of NAFLD and sets the stage for further liver damage.

### 4.2. Hepatic Response to Ectopic Fat Accumulation

**Figure 4:**
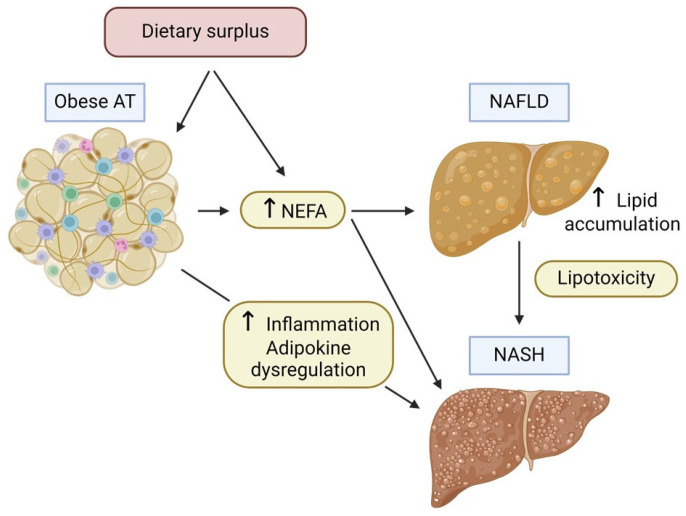
Adipose tissue—liver cross-talk in the progression of NAFLD/NASH associated with obesity. Abbreviations: NEFA, non-esterified fatty acid; NAFLD, non-alcoholic fatty liver disease; and NASH, non-alcoholic steatohepatitis.

In the context of NAFLD, hepatocytes are the primary cells affected by ectopic fat deposition. The accumulation of triglycerides in hepatocytes, initially a protective response to excess circulating free fatty acids, can lead to cellular stress and damage (Figure 4). This stress manifests in several ways:

- Lipotoxicity: Hepatocytes exposed to high levels of lipids, particularly saturated fatty acids and other toxic lipid species such as diacylglycerol (DAG) and ceramide, undergo lipotoxic stress. This can lead to cell dysfunction and apoptosis [50].
- Inflammatory Response: Ectopic fat in the liver can trigger an inflammatory response, attracting immune cells and producing pro-inflammatory cytokines. Moreover, chronic inflammation leads to significant histological changes in the liver, such as hepatocyte necrosis and apoptosis, neutrophil chemotaxis, activation of hepatic stellate cells, and production of Mallory bodies (aggregates of cytokeratin intermediate filaments). This plays a significant role in the disease’s development from basic steatosis to NASH and fibrosis. Furthermore, persistent inflammation may promote carcinogenesis and hence contribute to the progression of the disease to hepatocarcinoma (HCC) [51].
- Oxidative Stress: When lipid flow surpasses the capacities of both mitochondria and peroxisomes, respiratory oxidation becomes compromised, resulting in disturbances in lipid homeostasis, the generation of harmful metabolites, and an excess production of reactive oxygen species (ROS) [52]. These molecules precipitate oxidative stress and exacerbate hepatic necro-inflammatory processes, further aggravating mitochondrial damage. Moreover, ROS and oxidized low-density lipoproteins (LDL) can activate Kupffer and hepatic stellate cells, thus resulting in collagen deposition and secondary liver fibrosis [53,54].
- Endoplasmic Reticulum (ER) Stress: The accumulation of lipids can disturb ER function in hepatocytes, leading to unfolded protein response and further contributing to cellular stress and apoptosis [55].
- Altered Metabolism: Hepatocytes in a fatty liver have altered carbohydrate and lipid metabolism, often associated with insulin resistance. This metabolic dysfunction can exacerbate the accumulation of lipids in the liver and impair liver function [56].

In conclusion, the expandability of AT and its relationship with ectopic fat deposition, particularly in the liver, plays a crucial role in the development and progression of NAFLD. Understanding the mechanisms underlying AT expandability, hepatic lipid accumulation, and the subsequent cellular stress response is vital in addressing the growing burden of NAFLD in obesity.

## 5. Diagnostic Approaches and Biomarkers for Adipose Tissue Dysfunction

The escalating global prevalence of obesity presents a substantial public health challenge, given its established association with numerous diseases. Hence, there is a critical imperative to prevent, detect, and effectively manage obesity to mitigate its future health and economic ramifications. A pivotal initial stride toward this objective involves accurate diagnosis. In this regard, a myriad of diagnostic approaches have been proposed to classify obesity phenotypes and anticipate metabolic complications linked with obesity [57] (Table 1).

### 5.1. Anthropometric Parameters

Anthropometric parameters are widely used to diagnose obesity and its metabolic complications as they are non-invasive and cost-effective tests [58]. The body mass index (BMI) is the most widely used method to estimate the amount of body fat [59]. However, since BMI cannot detect regional variations in fat deposition, other measures able to capture abdominal obesity are also employed, such as waist circumference (WC) [60] or waist/hip ratio (WHR, waist circumference divided by the hip circumference) [61].

In general, none of these anthropometric parameters differentiate between fat and muscle mass, which have opposite health impacts, nor the contribution of subcutaneous or visceral fat [62,63]. Therefore, other methods considering adiposity and AT distribution are needed to classify obesity phenotypes correctly.

### 5.2. Imaging Techniques for Adipose Tissue Analysis

Due to the above-described limitations of classical obesity measures, other more complex techniques to assess body compartments have also been incorporated into obesity diagnosis [64]. Among them, advanced imaging techniques have revolutionized the diagnosis and understanding of obesity, particularly in assessing AT distribution [65]. Key methods include:

#### 5.2.1. Bioimpedance Analysis (BIA)

BIA utilizes electrical signals to estimate body composition, including fat mass and lean body mass. It is non-invasive and widely accessible, making it a standard tool for initial body composition assessments [66].

#### 5.2.2. Dual-Energy X-ray Absorptiometry (DXA)

DXA offers a detailed analysis of body composition, distinguishing between bone, lean, and fat mass. It is particularly effective for evaluating bone density but also provides accurate measurements of fat distribution.

#### 5.2.3. Computed Tomography (CT) and Magnetic Resonance Imaging (MRI)

These are considered the gold standard methods for quantitatively evaluating intra-abdominal AT distribution. CT and MRI allow for the precise differentiation between subcutaneous and visceral fat depots. They provide detailed images of fat distribution and are crucial for understanding the complex relationships between fat compartments and metabolic health [67,68]. The ratio of subcutaneous to visceral fat (sc/vis ratio), often determined through CT or MRI, is a critical indicator of metabolic health. This sc/vis ratio is important because the distribution of fat in the body—whether predominantly subcutaneous or visceral—has significant implications for metabolic and cardiovascular risk. A higher proportion of visceral fat is associated with a greater risk of metabolic syndromes, including insulin resistance, diabetes, and cardiovascular disease [69,70,71,72].

### 5.3. Circulating Biomarkers

Identifying biomarkers in human circulation that reflect the underlying biological mechanisms for the increased disease risk may be an alternative approach to characterize the relevant obesity phenotype. Several circulating molecules have been identified as obesity-associated biomarkers that can be classified into three main groups: adipokines, markers of the glucose-insulin pathway, and inflammatory markers [73,74,75].

#### 5.3.1. Adipokines

Adipokines have been proposed as biomarkers of obesity, as their production is often dysregulated in obese individuals and contribute to the pathogenesis of obesity-associated metabolic complications [76]. Numerous adipokines altered in the obese state have been identified in the past decades [77]. Higher leptin concentrations are observed in obese individuals, and leptin levels have been directly correlated with the percentage of fat mass [78,79]. Several studies have investigated associations of leptin levels with metabolic complications [80] and have shown that obesity-associated hyperleptinemia promotes hypertension [81], contributing to increased CVD risk [82,83].

By contrast, circulating adiponectin levels are inversely related to body weight, even though adiponectin is a protein synthesized and secreted predominantly by adipocytes into the peripheral blood. This inverse association is also observed with visceral fat accumulation [84]. The mechanism of this paradoxical relation remains unclear. Moreover, low circulating adiponectin concentrations are associated with a variety of diseases, including IR [85], T2DM, dyslipidemia [86], metabolic syndrome (MetS) [87,88], NAFLD [89], CVD [90] or atherosclerosis. At the same time, hyper-adiponectinemia is also associated with renal and pulmonary diseases [84].

The role of other adipokines, such as FABP-4 or visfatin, in obesity-related chronic disease risk is less well understood. For instance, a positive association between circulating FABP-4 and the risk of diabetes and heart failure has been suggested [91], while clinical studies have proposed a role of visfatin in inflammatory and atherogenic processes in various metabolic diseases, including T2DM and MetS [92].

#### 5.3.2. Markers of Glucose-Insulin Homeostasis

It is well known that obesity is associated with impaired glucose uptake and IR [93]. Therefore, different biomarkers related to insulin signaling have been investigated. For instance, fasting insulin and C-peptide, cleaved from proinsulin, have been shown to correlate positively with BMI [94]. Higher fasting insulin concentrations were associated with a higher risk of hypertension and coronary heart disease [95], while C-peptide has been shown to predict total and cardiovascular mortality in non-diabetic individuals [96,97]. Insulin metabolism is tightly linked with insulin-like growth factors (IGF), an evolutionarily conserved group of factors exerting long-term effects on growth [98]. IGF-1 has been proposed as a diabetes risk biomarker, as some studies showed a lower risk of glucose intolerance or T2DM in individuals with high versus low IGF-1 concentrations [99,100].

#### 5.3.3. Inflammatory Biomarkers

Obesity is associated with chronic low-grade systemic inflammation, which has been suggested to play a critical role in the pathogenesis of IR [101]. In AT of people with obesity, the secretion of cytokines such as TNF-α and IL-6 is upregulated, which stimulates the hepatic release of acute-phase proteins such as C-reactive protein (CRP) [102]. Due to the availability of standardized assays and its temporal stability, CRP is the most-studied inflammatory biomarker in relation to disease risk. Higher CRP concentrations have been associated with a higher risk of coronary heart disease, ischemic stroke, vascular and non-vascular mortality, as well as death from several cancers. However, CRP is rather unspecific [103].

### 5.4. Omics-Based Biomarkers

In this context, omics approaches have shown promise in improving our understanding of obesity and its diagnosis, as they integrate several data to uncover molecular patterns linked with the disease. Intensified efforts in omics research have been invested in the identification of genes (genomics), RNA (transcriptomics), and metabolites (metabolomics) linked to obesity. Further novel omic-based biomarkers include epigenomics, proteomics, glycomics, or microbiomics [104,105].

#### 5.4.1. Genomics

Genetic susceptibility to obesity is determined by the influence of multiple genetic variants [106,107]. Therefore, genome-wide association studies (GWAS) have become a useful tool to identify genetic variants critical in obesity that may serve as biomarkers [108]. A recent GWAS based on 700,000 individuals identified 941 near-independent SNPs associated with BMI [109]. Regarding body fat distribution, fewer variants have been associated. A large meta-analysis including 224 459 individuals identified and replicated 49 loci [110]. The strongest and most often replicated variants are within the Fat Mass and Obesity Associated (FTO) gene [111,112]. Some of the FTO genetic variants have been linked to appetite regulation [113,114], energy expenditure [115,116], or circadian rhythm [117] as well as to chronic diseases, including different types of cancer [118]. However, our understanding of how these genetic variants contribute to the development and progression of obesity remains incomplete [119]. Some other genetic variants linked to obesity are found within the genes of biomarkers described in the previous section, such as in the leptin, leptin receptor [120], or adiponectin [121].

#### 5.4.2. Transcriptomics

The transcriptome of adipocytes, from both subcutaneous and visceral WAT, has revealed more than a thousand genes whose expression is altered in obese as compared to lean individuals, as well as genes whose expression is correlated with the development or progression of metabolic complications [122].

Moreover, recent advancements in transcriptomic analysis have led to the exploration of single-cell transcriptomics in adipocytes, offering insights into the heterogeneity and dynamics of adipose tissue at a cellular level. This approach has facilitated the identification of specific gene signatures associated with adipocyte differentiation, lipid metabolism, and adipokine secretion, shedding light on the molecular mechanisms underlying adipose tissue dysfunction in obesity [123,124].

However, the limited availability of AT biopsies makes it difficult to use adipocyte transcriptome as a biomarker of metabolic status in clinical practice. As an alternative, peripheral blood transcriptome has also been studied to find biomarkers in obesity [125]. Some studies have correlated whole-blood mRNA levels with BMI, and the gene expression signatures pointed to key metabolic pathways involved in protein synthesis, enhanced cell death from pro-inflammatory or lipotoxic stimuli, enhanced insulin signaling, and reduced defense control against ROS [126].

Transcriptomic biomarkers include not only protein-coding RNAs (mRNAs), which represent less than 2% of the total genomic sequence, but also non-coding RNAs (ncRNAs) such as miRNAs and long ncRNAs (lncRNAs) [127]. miRNAs have emerged as promising biomarkers in obesity as they have shown to exert important regulatory roles in adipocytes [128,129], and some of them are released into the bloodstream [circulating miRNAs (cmiRNAs)] and, therefore, can be detected by minimally invasive methods. Several cmiRNAs with dysregulated expression in the plasma of people with obesity compared to lean have been identified, but further validation is needed to confirm their potential as biomarkers [130,131].

#### 5.4.3. Metabolomics

Alterations in many metabolites are associated with obesity, including higher plasma levels of branched-chain amino acids (BCAA) and aromatic amino acids (AAA), as well as lower plasma levels of glycine [132,133,134]. Interestingly, these biomarkers have also been linked to IR and a higher risk of T2DM [135,136]. An important branch of metabolomics with particular relevance in obesity research is lipidomics, as plasma lipids are mediators of metabolic dysfunction and obesity-related chronic diseases [137,138]. For decades, simple lipid profile analysis has been a fundamental tool in clinical practice to assess dyslipidemia [139,140]. In addition, various lipid species have been linked to obesity and its metabolic complications. For example, Mihalik et al. conducted lipidomic studies that revealed elevated levels of non-esterified fatty acids (NEFA) and short- and medium-chain acylcarnitines in obese individuals compared to lean subjects [141]. Moreover, subsequent investigations by Guasch-Ferré et al. [142] and Spiller et al. [143] corroborated these findings, indicating that many of these lipid markers are associated with an increased risk of type 2 diabetes mellitus (T2DM) independently of body mass index (BMI) and waist circumference (WC). This suggests that these lipid markers may enhance the predictive ability for the development of the disease.

### 5.5. Challenges and Future Directions

Despite the advancements in imaging and biomarker technologies, several challenges remain. The complexity of these techniques, the need for computerized processing, and the absence of established guidelines and thresholds for abnormal levels complicate their clinical applicability. Furthermore, factors such as ethnicity, age, sex, and fat-free mass might influence these levels, necessitating personalized approaches to obesity diagnosis and treatment.

In summary, integrating advanced imaging techniques and identifying specific biomarkers have significantly enhanced the understanding and diagnosis of obesity and its related metabolic disorders. These tools not only provide insights into body fat distribution and metabolic health but also offer opportunities for targeted interventions to improve overall health outcomes in individuals with obesity.

## 6. Therapeutic Strategies and Future Directions

### 6.1. Lifestyle, Surgical Options, and Pharmacological Interventions

Lifestyle changes, including diet and exercise, are fundamental in treating obesity and its metabolic complications. They are the initial recommendation for weight management and play a crucial role in improving metabolic health. Despite their significance, in many cases, lifestyle modifications alone may not suffice to reduce body weight significantly or halt the progression of obesity-related comorbidities.

Currently, bariatric surgery stands as the most effective and cost-saving intervention for obesity, particularly for those with severe obesity or obesity-related health complications. This surgical approach leads to substantial weight loss and improvement in obesity-related conditions such as type 2 diabetes, hypertension, and dyslipidemia. However, due to its complexity and associated risks, it is not a viable solution for the broader obesity pandemic [144].

There has been progress in pharmacological interventions targeting the energy balance regulatory system. Novel medications, such as those acting on the receptors of hormones released from the intestine, including glucagon-like peptide-1 (GLP-1) and glucose-dependent insulinotropic polypeptide (GIP), have shown promise in reducing appetite and food intake. These drugs are particularly useful for individuals who struggle to lose weight through lifestyle changes alone or those who have health issues directly related to obesity [145,146].

### 6.2. Innovative Therapies

In this context, the emergence of genetic engineering techniques and a deeper understanding of the molecular basis of obesity have led to novel precision medicine approaches targeting AT [147]. These innovative approaches include ADMSC-based therapy and gene therapy for obesity [148].

#### 6.2.1. ADMSC-Based Therapies

Transplantation of MSCs obtained from AT (ADMSCs) has been proposed as an alternative therapeutic strategy for obesity and its metabolic complications [149,150,151]. ADMSC can migrate to a wide range of tissues, including inflammatory and pathological sites, and possess immunomodulatory properties [152]. It has been reported that ADMSC transplantation improves AT inflammation by reducing pro-inflammatory cytokines such as interleukin 1β (IL-1β), IL-6, or TNF-α secretion [153,154,155]. It also restores glucose homeostasis by improving insulin sensitivity, as it contributes to the activation of insulin receptor substrate 1 (IRS-1)—serine/threonine kinase 1 (AKT)—GLUT4 pathway [156,157]. Moreover, ADMSC can differentiate into multiple lineages after transplantation. They can differentiate into insulin-producing cells (IPCs) and contribute to insulin production [158,159], as well as into hepatocyte-like cells (HLCs) and contribute to restoring liver function [154,160]. All these contribute to restoring the metabolic balance altered in obesity.

The results obtained in animal models have confirmed the effects of ADMSC therapy on weight loss, changes in AT composition, and improvement of related comorbidities such as diabetes or NAFLD [148,161].

#### 6.2.2. Genetic Modification of ADMSC: CRISPR/Cas9 Gene Editing

Genetic engineering of ADMSC has been proposed as a strategy to enhance the therapeutic potential of these cells and improve the clinical outcomes after transplantation. The CRISPR/Cas9 system, a groundbreaking tool in genetic engineering, offers the possibility of precisely editing genes associated with obesity and its comorbidities [162,163]. The efficiency of the CRISPR/Cas9 system has already been tested in murine MSC by targeting critical genes involved in adipocyte differentiation and function as *Pparg2*, *Prdm16*, *Zfp423*, or *Ucp1* [164], demonstrating that this system could efficiently manipulate gene expression in pre- and mature adipocytes in vitro. Regarding in vivo models, CRISPR/Cas9 technology was used to engineer human white adipocytes to display phenotypes similar to brown fat by targeting endogenous expression of uncoupling protein 1 (UCP1) [165] or nuclear-receptor-interacting protein 1 (NRIP1) [166]. Both studies showed the benefits of using CRISPR/Cas9 technology to treat metabolic complications. They demonstrated that it is a safe alternative, as ex vivo delivered Cas9 and sgRNA are entirely degraded by receptor cells after high-efficiency genomic modification without detectable off-target editing.

### 6.3. Future Directions

The future of obesity treatment lies in integrating these diverse approaches to develop comprehensive and personalized treatment plans. This could include a combination of lifestyle modifications, pharmacological interventions, surgical options, and advanced therapies like ADMSC-based treatment and genetic engineering. As our understanding of obesity’s molecular mechanisms expands, so will opportunities for innovative and effective treatments.

In summary, the treatment of obesity involves a multifaceted approach, encompassing lifestyle changes, surgical options, pharmacological therapies, and emerging technologies like genetic engineering and stem cell therapy. The evolution of these treatments, particularly in the realm of precision medicine, holds promise for more effective and personalized strategies to combat the obesity epidemic and its associated health risks.

## 7. Conclusions

The metabolic functions of AT are not confined to mere lipid storage; they extend to endocrine functions, immune responses, and systemic metabolic regulation. The understanding of WAT, particularly the differences between subcutaneous and visceral adipose tissues and the diverse cellular components within these tissues, is crucial in comprehending the complex role of AT in metabolic health and disease. Continued research into these areas is essential for developing targeted therapies for obesity and its related metabolic disorders, focusing on modulating the function and composition of AT to improve whole-body metabolic health.

## Figures and Tables

**Table 1:** Overview of diagnosis methods for obesity and its metabolic complications.

Category		Pros	Cons
Anthropometric parameters	BMI WC WHR	Non-invasive Cost-effective tests	Do not consider adiposity and AT distribution
Imaging techniques	BIA DXA CT MRI	Allow quantification of the fat volume and distribution High accuracy and reproducibility	Complex, require computerized processing Lack of guidelines and thresholds
Circulating biomarkers	Adipokines Insulin pathway Inflammatory markers	Non-invasive Cost-effective tests	Levels influenced by multiple factors Lack of guidelines and thresholds
Omics-based biomarkers	Genomics Transcriptomics Metabolomics Lipidomics	Integrate several data Allow more personalized diagnosis	Complex, require computerized processing Validation of its reproducibility is needed

## Data Availability

Not applicable.
